# Peer Review of “Influence of Mass Media on Italian Web Users During the COVID-19 Pandemic: Infodemiological Analysis”

**DOI:** 10.2196/34137

**Published:** 2021-10-18

**Authors:** Matheus Lotto

**Affiliations:** 1 Department of Pediatric Dentistry, Orthodontics, and Public Health Bauru School of Dentistry University of São Paulo São Paulo Brazil

**Keywords:** COVID-19, Google Trends, infodemiology, infoveillance, infodemic, media coverage, mass media influence, mass media, social media

*This is a peer-review report submitted for the paper “Influence of Mass Media on Italian Web Users During the COVID-19 Pandemic: Infodemiological Analysis.”*

## Round 1 Review

### General Comments

Thank you for providing me the opportunity of serving as a reviewer for this interesting manuscript [1]. The study aims to estimate the influence of Italian mass media on users’ web interests about COVID-19. It is original, well-written, and addresses an important research topic. Despite that, it presented several gaps that can be improved. Below are suggestions/doubts for the authors to consider.

### Specific Comments

1. The text presents some English errors. I recommend a grammar revision.

2. Introduction: Present a broader view on the subject to justify the study objectives. Moreover, avoid introducing methodological issues in this section.

3. Introduction: Why do people search for health-related information on the internet? How does the pandemic influence this behavior?

4. Introduction: What is the impact of infodemiology studies on public health? Also, what is the importance of infodemiological studies to combat the infodemic?

5. Introduction: What is Google Trends and what are its utility/advantages for infodemiological studies? The following studies may be helpful:

- Higgins TS, Wu AW, Sharma D, Illing EA, Rubel K, Ting JY, Snot Force Alliance. Correlations of online search engine trends with coronavirus disease (COVID-19) incidence: infodemiology study. *JMIR Public Health Surveill* 2020;6(2):e19702. doi: 10.2196/19702. PMID: 32401211
- Rovetta A, Bhagavathula AS. COVID-19-Related Web Search Behaviors and Infodemic Attitudes in Italy: Infodemiological Study. *JMIR Public Health Surveill* 2020;6(2):e19374. doi: 10.2196/19374. PMID: 32338613
- Lotto M, Aguirre PEA, Rios D, Machado MAAM, Cruvinel AFP, Cruvinel T. Analysis of the interests of Google users on toothache information. *PLoS One* 2017;12(10):e0186059. doi: 10.1371/journal.pone.0186059. PMID: 29049315
- Mavragani A, Ochoa G. Google Trends in infodemiology and infoveillance: methodology framework. *JMIR Public Health Surveill* 2019;5(2):e13439. doi:10.2196/13439. PMID:31144671
- Nuti SV, Wayda B, Ranasinghe I, Wang S, Dreyer RP, Chen SI, Murugiah K. The use of Google Trends in health care research: a systematic review. *PLoS One* 2014;9(10):e109583. doi: 10.1371/journal.pone.0109583. PMID: 25337815

6. Introduction: The third aspect proposed for investigation is not adequate. The authors go beyond the main scope by introducing a “fake news” analysis. The authors did not define what “fake news” is, and the analysis made is too superficial for the proposed outcome. I recommend that this step be better planned and analyzed, being described in a future manuscript.

7. Introduction: Question R4 has already been widely discussed in the literature and can be removed. If necessary, this aspect can be presented in the *Discussion* section.

8. Methods: The data collection is confusing and needs to be better described. Why were these platforms chosen? What are the main media in Italy and which ones have been selected? Describe in detail how data were collected on each platform.

9. Methods: Does analyzing search results on platforms really show the influence of the media? Why was a qualitative study not proposed?

10. Methods: What is the infodemic scale? Present it in detail.

11. Methods: Is the normalization of media data on the same scale as Google Trends correct? Since the absolute Google Trends data is blind, it doesn’t seem like a valid comparison to me.

12. Methods: What test was done to assess seasonality?

13. Results: Avoid discussing the results in advance, as in “Evidence supporting causation.”

14. Discussion: Only media influence is discussed about Google Trends data. What other factors might influence the findings? Why do people search for information related to COVID-19 during the pandemic?

15. Discussion: Compare the findings with previous literature. Several studies have investigated users’ COVID-19–related interests on Google Trends, including in Italy.

16. Discussion: Why are technical terms little used? Where does the Italian population learn about the pandemic? How do eHealth literacy and media literacy influence this process?

17. Discussion: Include a *Practical Implications* subsection.

## Round 2 Review

### General Comments

Thank you for reviewing the manuscript based on my comments. In general, the suggestions were satisfactorily answered, and the quality of the paper has improved considerably.

## References

[1] Rovetta A, Castaldo L (2021). Influence of mass media on Italian web users during the COVID-19 pandemic: infodemiological analysis. JMIRx Med.

